# Serum-Resistant Ternary DNA Polyplexes for Suicide Gene Therapy of Uterine Leiomyoma

**DOI:** 10.3390/ijms25010034

**Published:** 2023-12-19

**Authors:** Anna Egorova, Sofia Shtykalova, Marianna Maretina, Svetlana Freund, Alexander Selutin, Natalia Shved, Sergei Selkov, Anton Kiselev

**Affiliations:** 1 Department of Genomic Medicine Named after V.S. Baranov, D.O. Ott Research Institute of Obstetrics, Gynecology and Reproductology, Mendeleevskaya Line 3, 199034 Saint-Petersburg, Russia; egorova_anna@yahoo.com (A.E.); sofia.shtykalova@gmail.com (S.S.); marianna0204@gmail.com (M.M.); svetafreundmax@gmail.com (S.F.); natashved@mail.ru (N.S.); 2 Department of Immunology and Intercellular Interactions, D.O. Ott Research Institute of Obstetrics, Gynecology and Reproductology, Mendeleevskaya Line 3, 199034 Saint-Petersburg, Russia; a_selutin@yahoo.com (A.S.); selkovsa@mail.ru (S.S.)

**Keywords:** uterine leiomyoma, DNA delivery, peptide-based carriers, gene therapy, thymidine kinase, integrins, RGD, organotypic model, anionic coating, serum resistance

## Abstract

Uterine leiomyoma (UL) is a prevalent benign tumor in women that frequently gives rise to a multitude of reproductive complications. The use of suicide gene therapy has been proposed as a highly promising method for treating UL. To achieve successful gene therapy, it is essential to develop carriers that can efficiently transport nucleic acids into targeted cells and tissues. The instability of polyplexes in blood and other biological fluids is a crucial factor to consider when using non-viral carriers. In this study, we present serum-resistant and cRGD-modified DNA complexes for targeted delivery genes to UL cells. Ternary polyplexes were formed by incorporating cystine-cross-linked polyglutamic acid modified with histidine residues. We employed two techniques in the production of cross-linked polyanionic coating: matrix polymerization and oxidative polycondensation. In this study, we investigated the physicochemical properties of ternary DNA complexes, including the size and zeta-potential of the nanoparticles. Additionally, we evaluated cellular uptake, toxicity levels, transfection efficiency and specificity in vitro. The study involved introducing the HSV-TK gene into primary UL cells as a form of suicide gene therapy modeling. We have effectively employed ternary peptide-based complexes for gene delivery into the UL organtypic model. By implementing in situ suicide gene therapy, the increase in apoptosis genes expression was detected, providing conclusive evidence of apoptosis occurring in the transfected UL tissues. The results of the study strongly suggest that the developed ternary polyplexes show potential as a valuable tool in the implementation of suicide gene therapy for UL.

## 1. Introduction

Gene therapy represents a groundbreaking therapeutic approach to effectively treat or alleviate inherited and acquired human diseases that do not respond well to standard treatments [1]. Considerable effort has been focused on the selection of diseases that can be most suitably targeted by gene therapy approaches. Uterine leiomyoma (UL) is the most common benign tumor for women of reproductive age and can cause abnormal bleeding, pelvic pain, pregnancy complications, and infertility [2]. The conventional surgical method for treating UL is becoming less appealing to women who are considering having children in the future [3]. Women with symptomatic leiomyomas who desire to preserve their fertility face a lack of viable options. The impact of myomectomy on future fertility remains disputed, as concerns arise regarding potential obstruction due to postoperative adhesions [4]. Alternatives such as myolysis and uterine artery embolization have their drawbacks and are not suitable for women planning pregnancies. Furthermore, the long-term use of hormonal treatment is limited due to the grave side effects it causes [5,6]. The treatment of UL is still the subject of controversy due to the lack of an effective and organ-preserving approach. The advancement of molecular technologies and the discovery of new data regarding the molecular aspects of disease pathogenesis have led to a rise in the availability of organ-preserving methods and drugs that effectively suppress tumor growth and development [3,7,8,9]. It can be suggested that uterine leiomyoma seems to be a worthy candidate for gene therapy application, as Myomatous nodes can be easily located and treated using various endoscopic techniques. Moreover, the relative lack of metastasis renders this condition a perfect candidate for localized gene therapy. One notable factor is that this treatment does not affect patients’ fertility or hormonal systems. However, final confirmation of this aspect can only be obtained through clinical trials [10,11]. 

A prospective approach for gene therapy of UL as tumor disease is suicide gene therapy. The concept behind suicide gene therapy involves the introduction of a specific gene, known as a “suicide” gene, into tumors. This gene enables the conversion of a prodrug into a potent and lethal drug [12]. The HSV-TK/GCV system is highly regarded as one of the most promising “suicide” systems. The expression of the *HSV-TK* gene leads to the synthesis of viral thymidine kinase, which transforms GCV into ganciclovir monophosphate, thus enabling its phosphorylation by cellular kinases to the triphosphate. Ganciclovir triphosphate, which is similar to cellular deoxyguanosine triphosphate, integrates into DNA and effectively halts DNA replication, leading to the demise of tumor cells [13]. The attractiveness of this approach is further enhanced by well-known “bystander effect”, made possible by the abundant presence of gap junctions in leiomyoma cells, in comparison to normal myometrium [14,15]. During intercellular interactions, phosphorylated ganciclovir is transferred to neighboring cells, and this phenomenon is closely linked to this process [16]. It is important to note that the suicide gene therapy that uses the HSV-TK/GCV system has already been employed in gene therapy research of uterine leiomyoma [7,10,17,18,19,20,21,22,23].

In order to achieve successful gene therapy, it is crucial to create highly efficient carriers that can effectively deliver nucleic acids into specific cells and tissues. There are numerous studies that concentrate on harnessing viral particles as vectors for enhanced transfection efficiency. Nevertheless, certain limitations related to viral vectors, such as toxicity, immunogenicity, and particle scaling restrictions, have triggered the advancement of secure and effective non-viral gene delivery carriers [24,25]. Cationic polymers have undergone extensive research as a safer alternative to viruses for gene therapy applications. There are several advantages to consider when it comes to these carriers. One notable advantage is that they do not trigger a specific immune response. Additionally, they have a higher capacity to transport nucleic acid. In addition, the carrier can be modified to enhance its structure and composition. Lastly, they hold great potential for large-scale production [26].

Peptide-based carriers exhibit several favorable characteristics among cationic polymers. Nucleic acids can be condensed through the use of electrostatic interactions, with the assistance of positively charged amino acids such as lysine, arginine, or ornithine. Additionally, modifying the carriers with histidine or fusogenic peptide domains can improve their ability to escape the endosome [27]. Implementing bioreversible crosslinking strategies can provide a means to stabilize the polyplexes. Furthermore, by incorporating a ligand part, receptor-mediated gene delivery can be achieved [28]. Moreover, peptide carriers obtained by solid-phase peptide synthesis possess a sequence with defined structure and monodispersity, which has allowed a precise structure-activity relationship to become established in transfection studies [29]. Previously, we developed and characterized in detail arginine-cysteine-histidine-rich peptide carriers for nucleic acid condensation, stabilization and endosomal escape. The carriers were additionally modified with the cyclic RGD (arginine–glycine–aspartic acid) ligand to provide efficient and specific gene delivery to uterine leiomyoma cells with overexpressed αvβ3 integrins on them. After evaluating the cell proliferative activity and apoptosis, it was found that the obtained DNA polyplexes successfully enabled the suicide gene therapy of primary leiomyoma cells in vitro [20,21].

One crucial factor to consider when using peptide carriers is the instability of polyplexes in blood and other biological fluids. In order to obtain efficient polyplexes, it is common practice to use an excess of cationic carriers, leading to the formation of positively charged polyplexes in most instances. Following in vivo gene delivery, both serum proteins and the extracellular matrix have the capability to unpack the nucleic acid and carrier complexes [30]. When polyplexes are injected intravenously, they interact with negatively charged serum proteins. This interaction often leads to the aggregation or dissociation of the complexes. Furthermore, the nucleic acids within these complexes can also undergo degradation by serum nucleases; as a result, this process inhibits transfection [31,32]. In addition, the intravenous administration of positively charged polyplexes can potentially trigger the activation of various cell mediators of toxicity, including macrophages, monocytes, and neutrophils [33]. One suggested way to overcome this obstacle is by administering localized injections at the affected areas. One important characteristic of uterine leiomyomas is their abundance of extracellular matrix. Unfortunately, this abundance hinders the success of gene therapy for the disease when utilizing cationic polyplexes [34]. Thus, regardless of the means of administration, polyplexes stability issues should be addressed to ensure efficient transfection of uterine leiomyoma tissue.

The charge of the cationic polyplexes can be effectively neutralized, enabling them to evade biological fluids and greatly enhance their in vivo biodistribution [35]. For this purpose, several strategies could be offered. The most commonly used approach is the chemical modification of the carrier with hydrophilic-non-ionic polymer–poly (ethylene glycol) (PEG). It was shown that serum had a relatively weak inhibitory effect on the transfection efficacy of PEG-modified polyplexes [36]. Ibaraki et al. discovered that administering MPEG-PCL-CH_2_R_4_H_2_C/siRNA micelles intravenously in mice leads to remarkable therapeutic results for pulmonary melanoma metastasis, as well as inflammatory diseases like rheumatoid arthritis and ulcerative colitis. This study conclusively demonstrated the effectiveness of methoxy-PEG-modified nanomicelles as carriers fordelivering therapeutic nucleic acids systemically and accumulating them at inflammatory sites [37,38,39]. Another approach to neutralize cationic polyplexes involves the creation of ternary complexes through the addition of anionic polymers, specifically glycosaminoglycans like chondroitin sulfate (CS), hyaluronic acid (HA), and others [40]. Iwanaga and colleagues conducted a study of the intravenous injection of a nonviral gene system that was based on dendrigraft poly-L-lysine. They found that high gene expression in vivo was only observed when CS was added [40]. One popular strategy for cationic polyplexes shielding is obtaining ternary polyplexes with negatively charged polyglutamic acids (PGAs). PGAs have low toxicity, hydrophilicity, non-immunogenicity, biocompatibility, biodegradability, and can easily interact with positively charged complexes. Moreover they can decrease the toxicity of cationic polyplexes without reducing their transgene expression efficiency [41,42,43]. Kurosaki et al. reported that, after intravenous injection, γ-polyglutamic acid-coated cationic complexes of DNA and poly-L-arginine or poly-L-lysine showed high gene expression in the spleen [44]. Kodama et al. demonstrated that after γ-PGA-coated dendrigraft poly-L-lysine-based polyplexes were intravenously injected into mice, they displayed high transfection efficiency (mainly in the spleen) and negligible liver toxicity [45,46]. Importantly, DNA vaccination using the ternary vehicles suppressed the growth of melanoma and lung metastasis in mice by activating the systemic immunity, proving the efficiency and safety of ternary PGA-coated polypeptide complexes for clinical gene therapy [46].

Herein, we developed serum-resistant ternary DNA complexes for gene delivery to the UL cells and tissues. To enhance DNA binding and facilitate endosome escape, we utilized polycondensed peptide carriers containing arginine, histidine, and cysteine, which have been extensively studied before [21,47]. The addition of bioreducible-cystine- cross-linked-poly-L glutamic acid, modified with histidine residues for additional endosome buffering, resulted in the formation of ternary polyplexes. We used two different methods to create cross-linked PGAs: matrix polymerization, which involved the formation of interpeptide disulfide bonds during DNA complexation; and oxidative polycondensation using a catalytic agent–dimethyl sulfoxide (DMSO) [48]. For targeted gene delivery to αvβ3 integrin-expressing cells we modified either core cationic peptide oligomer or PGA oligomer with cyclic RGD ligand during the polycondensation reaction. The physicochemical properties of the ternary DNA-complexes (the size, zeta-potential of the nanoparticles and the efficiency of DNA complex formation), as well the assessment of the complexes’ transport into cells, toxicity, transfection efficiency and specificity, were studied in vitro. Further research was conducted into the most promising complexes to determine their effectiveness as gene therapeutics for uterine leiomyoma treatment. The suicide gene therapy modeling encompassed the transfer of the HSV-TK gene into primary cells derived from myomatous nodes of patients with UL. This was followed by a thorough evaluation of the cells’ proliferative activity. Following that, we aimed to showcase the effectiveness of in situ gene delivery by utilizing an organotypic model of UL [49]. We successfully utilized ternary peptide-based complexes to deliver marker and therapeutic genes into UL tissue. Through modeling of in situ suicide gene therapy, we were able to detect the expression of apoptosis markers, indicating the apoptosis of the transfected UL tissues.

## 2. Results and Discussion

Cationic peptide carriers represent a promising and extensively studied group of non-viral genetic delivery vectors. Their in vivo instability, however, poses a fundamental challenge to the field [50,51]. The inclusion of polyanionic component to DNA/cationic peptide complexes may prevent a serum-mediated inhibition of transfection, both in vitro and in vivo [39,40,46]. In this study biodegradable cystine-cross-linked-poly-L glutamic acid was added to the pDNA/arginine-histidine-cysteine-rich peptide-based polyplexes. Similar polyanionic compounds can be modified with ligands to target cell receptors and effectively carry out specific delivery without loss of their properties [35,52]. However, this addition resulted in alterations to the physicochemical properties of the peptide-based complexes, including changes in surface charge and size. Moreover, it greatly improved the transfection capacity of these complexes in a serum-containing medium.

### 2.1. Design of the Carriers

In this study, we utilized R6p and R6p-cRGD cationic carriers, which were obtained from our previous research, to form complexes with plasmid DNA [21]. The previous studies have demonstrated the remarkable effectiveness of the carrier’s composition, which consists of a combination of arginine, histidine, and cysteine. This combination has shown to effectively facilitate cellular uptake, escape from endosomes, and release intracellular DNA [47,53]. We successfully modified the R6p carrier by adding cRGD at a ratio of one ligand molecule per two R6 monomers, as previously described [21]. This modification allowed us to achieve targeted gene delivery into cells expressing αvβ3 integrin. For this study, we have developed an enhanced version of the anionic cysteine-flanked histidine-rich E6 peptide by incorporating glutamic acid. This modification enhances the peptide’s ability to interact with cationic complexes, disrupt endosomes, and effectively release DNA inside cells. In addition, we successfully synthesized a variety of polymers, including the E6 peptide through matrix polymerization, as well as the E6p and E6p-cRGD polymers through oxidative polycondensation. The E6p-cRGD polymer was prepared following a similar method to the R6p-cRGD polymer. Carriers’ design is summarized in Table 1. We gradually increased the anionic moiety in the composition of the polyplexes by adding E6, E6p, or E6p-cRGD polymers to DNA/R6p or DNA/R6p-cRGD complexes. Different ternary complexes were formed at various P/N/C (Phosphate/Nitrogen/Carboxyl) ratios, including DNA/R6p/E6, DNA/R6p-cRGD/E6, DNA/R6p/E6p, DNA/R6p-cRGD/E6p and DNA/R6p/E6p-cRGD.

### 2.2. Assessment of pDNA Binding and Relaxation of Polyplexes by Dextran Sulfate 

The process of DNA binding to cationic carriers involves the self-assembly of relaxed DNA into compact nanoparticles [54]. Adding anionic components to the complexes has a significant impact on the density of the particles and other physicochemical characteristics, which directly affects their functional properties. We optimized the formulation of ternary polyplexes in our study using both the EtBr exclusion assay and the dextran sulfate displacement assay. Previously, we have shown that DNA/R6p and DNA/R6p-cRGD complexes formed at N/P ratios 8/1 and 12/1 are colloidally stable and can provide full DNA binding and protection. Thus, these complexes have been chosen for modification with anionic components [21].

**Figure 1:**
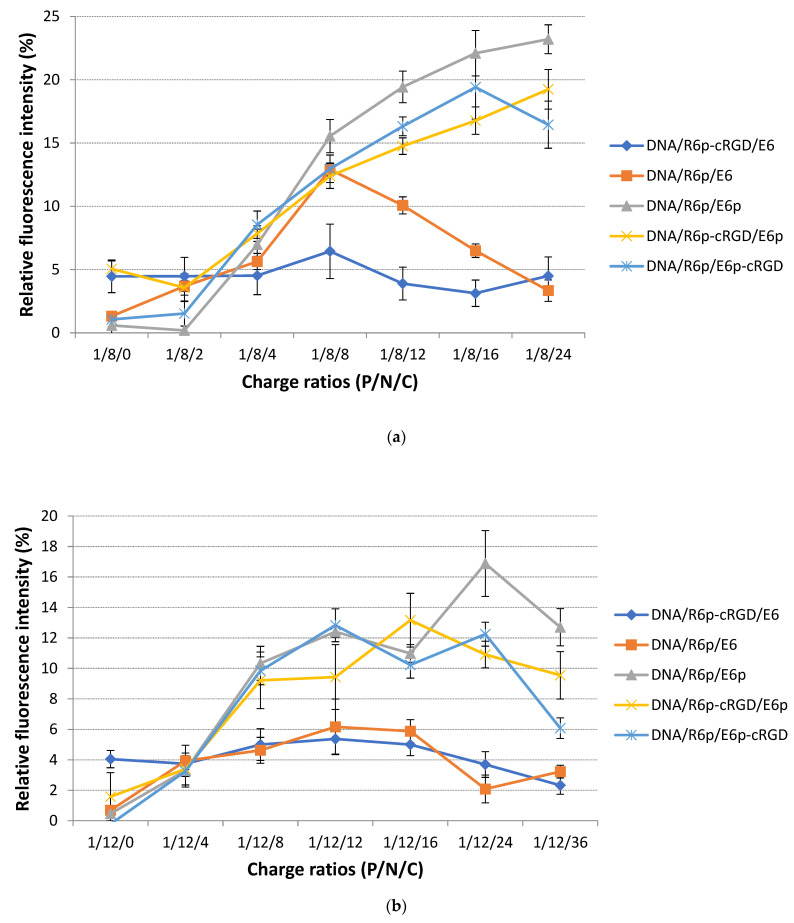
EtBr exclusion assay of DNA/R6p and DNA/R6p-cRGD complexes formed at 8/1 (**a**) and 12/1 (**b**) N/P ratios after addition of E6, E6p or E6p-cRGD anionic polypeptides at different C/P. Values are the mean ± SD of the mean of triplicates.

**Figure 2:**
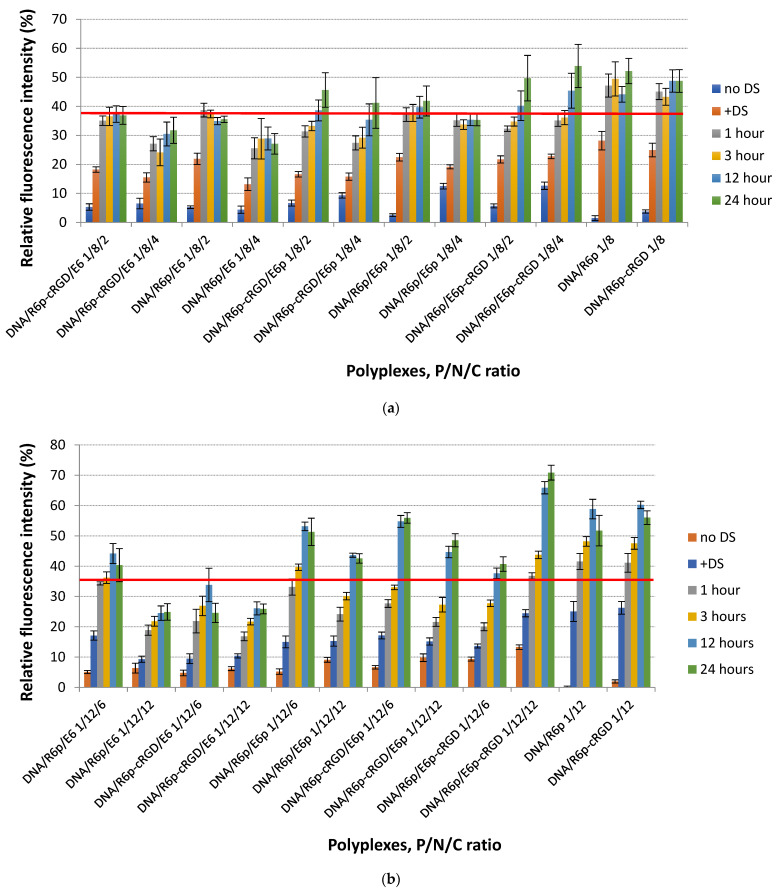
Relaxation of ternary DNA-peptide complexes formed with R6p or R6p-cRGD cationic carriers and E6, E6p of E6p-cRGD anionic component at 8/1 (**a**) and 12/1 (**b**) N/P ratio during 24 h of dextran sulfate (DS) treatment in three-fold charge excess relative to cationic moiety. Values are the mean ± SEM of the mean of triplicates. The red line indicates a 35% level of relaxation.

The formation of polyplexes and effect of anionic polypeptides on the density of complexes were registered by EtBr exclusion assay. The fluorescence of intercalated EtBr with naked pDNA was assessed as 100%. The relative fluorescence intensity was decreased almost to zero level when cationic peptides were added to DNA at N/P ratio 8/1 and 12/1. This result has confirmed the high efficiency of pDNA condensation (Figure 1). The addition of E6p or E6p-cRGD polycondenced peptides to DNA/R6p and DNA/R6p-cRGD polyplexes resulted in slight increase in EtBr fluorenscence intensity, especially at C/P ratio higher than 12/1 for complexes formed with 8/1 N/P ratio, and higher than 24/1 for complexes formed at 12/1 N/P ratio. Increasing the charge of the anionic component to cationic carriers by twice or more resulted in the registration of approximately 20% relaxation in DNA-peptide complexes. In contrast, the density of ternary polyplexes remains unaffected by the E6 peptide. The difference in density of complexes is assumed to be dependent on the structure of DNA-complexes, specifically those formed with template-polymerized and oxidatively condensed anionic peptides [47]. Moreover, when a polyanionic component is added to cationic DNA/peptide complexes, it can create unique structures while still maintaining the interaction between DNA and the cationic molecules. Remarkably, under physiological conditions, the ternary complexes remained intact even when the N/C ratio was less than one [33]. The results obtained from dextran sulfate-mediated relaxation could shed light on the variations in density between ternary polyplexes formed using different polymerization methods (Figure 2). The utilization of dextran sulfate as a competitive polyanion provides a valuable tool that can be used to study the complex interaction between ternary polyplexes and glycosaminoglycans, the primary extracellular anionic components, and mRNA, the primary intracellular anionic component.

Glycosaminoglycans (GAGs), such as heparan sulfate and chondroitin sulfate, play a crucial role in providing a strong negative charge on the surface of cells [55]. It has been proposed that they have a significant influence on cationic gene delivery systems [56]. On the one hand, GAGs can enhance the efficacy of targeted gene delivery via electrostatically binding to cationic polyplexes and acting as receptors [57]. On the other hand, the excessive attachment of the polyplexes to the GAGs on the cell surface has the potential to disrupt their stability [58]. The rate of destabilization of ternary complexes can be decreased by adding an anionic moiety to cationic DNA-peptide complexes. The application of dextran sulfate to DNA-peptide complexes led to the discovery that the permeability of DNA/R6p-cRGD and DNA/R6p complexes to EtBr dye increased at a considerably faster rate compared to complexes containing anionic polypeptides (Figure 2). This suggests that the incorporation of negatively charged components into polyplex compositions enhances their ability to resist the effects of external polyanions. It is essential to note that the ternary polyplexes created with the template-polymerized anionic peptide E6 displayed higher resistance to polyanions when compared to the oxidatively-condensed E6p and E6p-cRGD polymers. The template-polymerized E6 peptide is expected to have the ability to form disulfide bonds within individual complexes, as well as between multiple complexes. This results in the formation of aggregates that are resistant to relaxation. Nevertheless, polyanion-mediated relaxation has a dual effect on non-viral transfection. It can enhance DNA release from the complexes in the cytoplasm, which results in augmentation of transfection process [59]. However, increased resistance of the polyplexes can impede the intracellular DNA release, consequently diminishing its subsequent expression and therapeutic effectiveness [60,61].

### 2.3. Disulfide Bonds Formation during Template Polymerization

To resolve the stability dilemma of polyplexes, which need to be stable in the bloodstream but dissociate inside cells, researchers have developed polymers with cleavable backbones, such as cystine-crosslinked polypeptides. These polymers effectively mitigate the DNA binding of the polyplexes by responding to intracellular stimuli, such as glutathione (GSH) [47,62]. 

**Figure 3:**
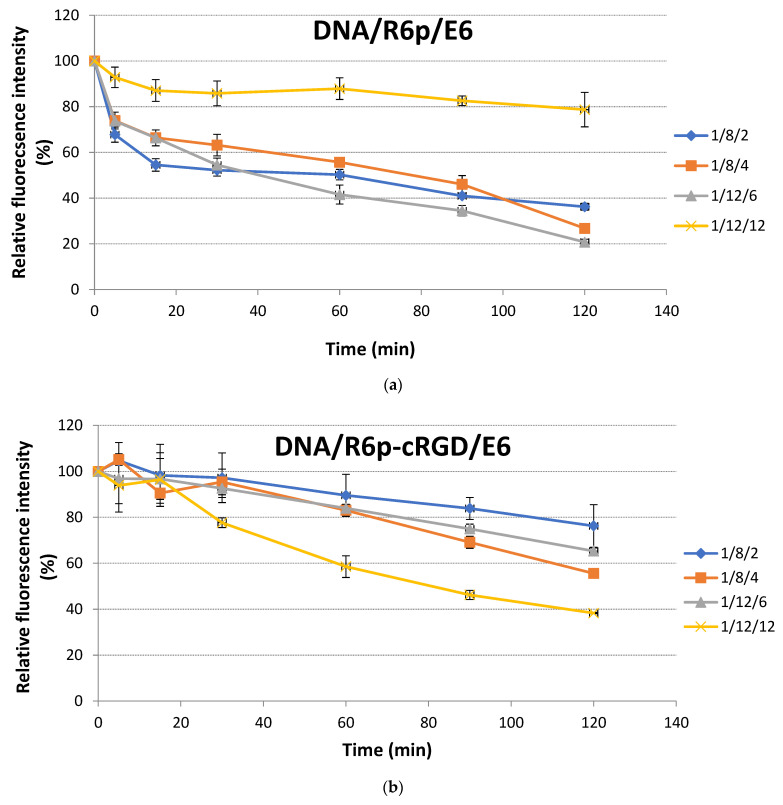
Monitoring of disulfide bonds formation during template polymerization of E6 peptide added to DNA/R6p (**a**) or DNA/R6p-cRGD (**b**) polyplexes formed at different P/N/C ratios. 100% of absorbance intensity corresponds to free E6 peptide stained with Ellman’s reagent. The data are shown as the mean ± SD.

The polymers R6p, R6p-cRGD, E6p, and E6p-cRGD obtained through oxidative polycondensation were found to have no more than 5% of free thiol groups in their composition. This suggests that the involvement of thiol groups in further complex formation is relatively low. After template polymerization, the quantity of free thiol groups may vary, suggesting a varying level of involvement of disulfide groups in DNA complexation. To accurately measure the relative quantity of thiol groups in the template-polymerized E6 peptide, which was added to DNA/R6p or DNA/R6p-cRGD polyplexes, we implemented Ellman’s assay. This method allowed us to closely monitor the formation of disulfide bonds over a period of 2 h (Figure 3). The relative quantity of thiol groups in E6 peptide added to DNA/R6p polyplex at 1/8/2 and 1/8/4 charge ratios was approximately 36% and 27% after 120 min of incubation, respectively. For DNA/R6p polyplexes formed at P/N/C ratios 1/12/6, an 1/12/12 addition of E6 peptide resulted in a decrease of free thiol groups of up to 21 and 78%, respectively (Figure 3a). In ternary DNA/R6p/E6 polyplexes at a charge ratio of 1/12/12, the majority of thiol groups remain unreacted, with only 22% participating in the formation of disulfide bonds. The presence of a large quantity of anionic E6 peptide may have a significant effect on intermolecular repulsion, potentially resulting in a delay in the formation of interpeptide disulfide bonds. However, when examining alternative charge ratios for DNA/R6p/E6 polyplexes, it was observed that the majority of thiol groups were oxidized. On the contrary, the inclusion of the E6 peptide in DNA/R6p-cRGD polyplexes led to a less efficient formation of disulfide bonds. We suggest that including an uncharged cyclic RGD ligand in the cationic carrier composition could potentially influence the stereochemistry of complex formation. This is because the cyclic ligand, with its less flexible conformational structure compared to a linear ligand, might hinder the formation of disulfide bonds [63]. It has been discovered that the formation of approximately 60% of the disulfide bonds can only be achieved by adding a substantial amount of E6 peptide to DNA/R6p-cRGD polyplex formed at P/N/C ratio 1/12/12 (Figure 3b).

### 2.4. Measurement of Size and Z-Potential of Ternary Polyplexes

**Figure 4:**
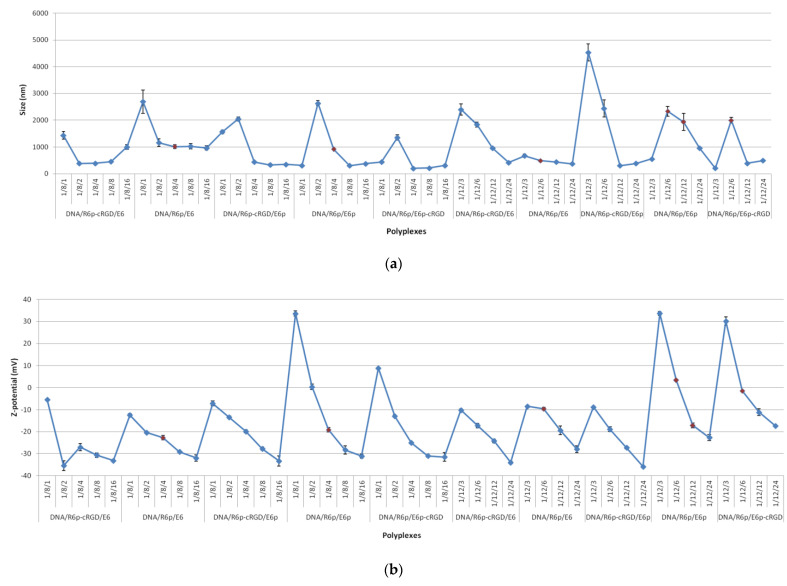
Size (**a**) and ʐ-potential (**b**) of the ternary DNA-peptide polyplexes. The data are shown as the mean ± S.D. The red circles mark the polyplexes with the highest transfection activity (see Figure 5).

The size and zeta-potential of non-viral gene delivery vectors play a crucial role in determining their toxicity and transfection efficiency [45]. DNA-polyplexes, depending on their size, utilize different intracellular trafficking pathways to entry inside the cells. Particles with a size smaller than 200 nm can be internalized through both clathrin-dependent and caveolin-dependent pathways, as well as through pathways that are independent of these two mechanisms [64]. Actin-dependent membrane protrusion is responsible for the internalization of larger complexes into spacious vacuoles, culminating in the formation of remarkably large macropinosomes ranging from 0.5 to 10 µm in size [65]. The DNA/R6p and DNA/R6p-cRGD polyplexes were determined to have a compact size ranging from 150 to 200 nm when measured in a HBM buffer [21]. However, the complexes formed with E6, E6p, or E6p-cRGD polymers exhibited a larger size ranging from 200 to 4600 nm, depending on the specific type of ternary polyplexes (Figure 4a). The polydispersity index (PDI) values varied from 0.2 up to 0.4 for smaller polyplexes, whereas bigger polyplexes had PDI values in the range of 0.4–0.8. As expected, in most cases, increasing the concentration of polyglutamic acid led to a decrease in the size of the polyplexes. This is likely because it promotes the formation of more dense complexes. The attachment of polyplexes to the cell membrane seems to be most affected by the particles’ surface charge [66]. The positive surface charge of the polyplexes may promote cellular uptake via electrostatic interaction with the negatively charged cell membrane and thereby enhance gene expression [67]. The DNA/R6p and DNA/R6p-cRGD polyplexes are positively charged with ʐ-potential from +30 to +36 mV, as shown previously [21]. However, the presence of positively charged particles in the bloodstream and biological fluids can lead to their rapid opsonization and clearance by macrophages. In addition, the display of a positive surface charge by these particles could have negative consequences, as it may lead to cytotoxic effects. Modifying the surface of these complexes with an anionic coating has the potential to give us control over opsonization and cytotoxicity, while also preventing their clearance by altering the surface charge [68]. We have observed that increasing the amount of polyglutamic acid causes the zeta potential to gradually decrease to −35 mV (Figure 4b).

### 2.5. Assessment of Cytotoxicity

The cytotoxicity greatly depends on the charge density of polyplexes. It is well-established that positively charged polyplexes are known to cause cell membrane toxicity [69]. Furthermore, exceeding the charge neutral point in polyplexes by an excess of cations increases cytotoxicity due to their non-specific aggregation with components present in the extracellular fluid [70]. There are multiple strategies for reducing the cytotoxicity of polyplexes. A potentially effective method could involve incorporating negatively charged PGA into cationic polyplexes. Previous studies have demonstrated that PGA has the ability to alleviate the toxicity associated with cationic compounds [44,45]. An additional method for reducing cytotoxicity is through the modification of polymers using bioreducible disulfide bridges [47]. Here, we have combined these two approaches to enhance the development of polyplexes. The cytotoxicity of the studied ternary polyplexes was assessed 24 h after their addition by means of the Alamar Blue assay. PANC-1 cells were transfected with the complexes formed at different P/N/C ratios (Figure S1). The assay clearly indicated that none of the polyplexes examined significantly compromised the viability of the PANC-1 cells. This demonstrates that these complexes are indeed biocompatible with the cell membranes.

### 2.6. Transfection Studies

**Figure 5:**
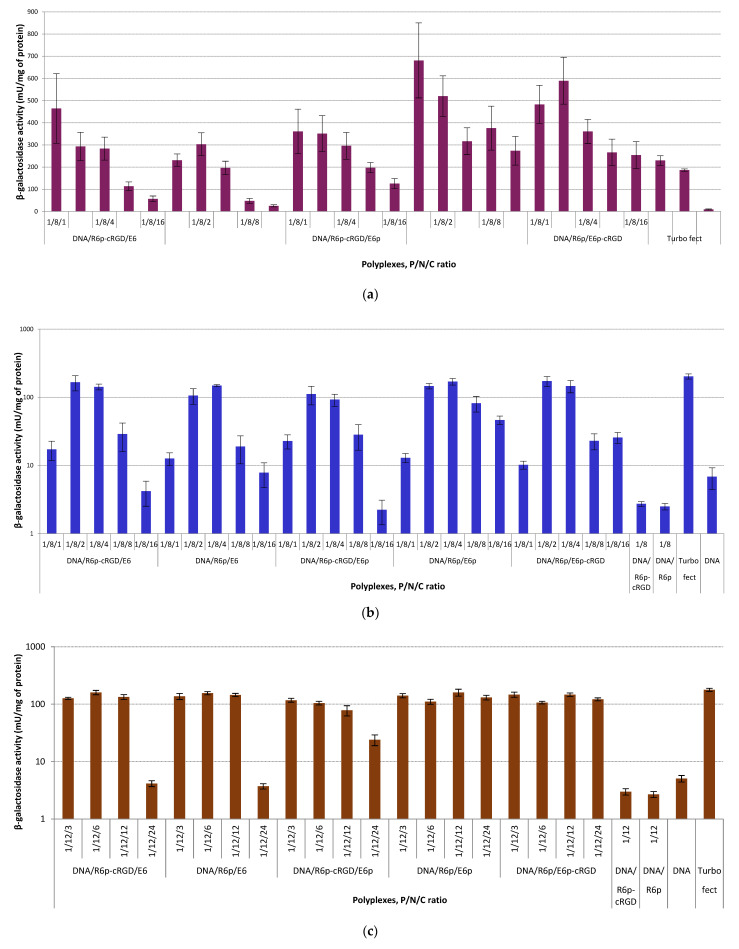
Transfection efficacy studies in the PANC-1 cells: (**a**) the cells were transfected in serum-free medium with DNA/R6p and DNA/R6p-cRGD complexes at charge ratio of 8/1, ternary polyplexes with different C/P ratios, naked plasmid pCMV-lacZ, Turbofect/DNA and PEI/DNA complexes; (**b**) the cells were transfected in serum-supplemented medium with DNA/R6p and DNA/R6p-cRGD complexes at charge ratio of 8/1, ternary polyplexes with different C/P ratios, naked plasmid pCMV-lacZ, and Turbofect/DNA; (**c**) the cells were transfected in serum-supplemented medium with DNA/R6p and DNA/R6p-cRGD complexes at charge ratio of 12/1, ternary polyplexes with different C/P ratios, naked plasmid pCMV-lacZ, and Turbofect/DNA. Values are the mean ± SD of the mean of triplicates.

The transfection efficiency of the ternary DNA-polyplexes at different P/N/C ratios was evaluated in αvβ3 integrin-positive PANC-1 cells by using both FBS-free and FBS-supplemented medium, with pCMV-lacZ as a reporter gene plasmid. Initial experiments were carried out in serum-free medium at DNA/R6p and DNA/R6p-cRGD ratio of 1/8. DNA/PEI and DNA/Turbofect complexes were used as positive controls (Figure 5a). Polyglutamic acid-rich polypeptides obtained after oxidative polycondensation and after matrix polymerization were formed at C/P ratios that varied from 1/1 to 16/1. The ternary complexes that showed the highest effectiveness were the ones with a minimal quantity of polyanions. The complexes formed with R6p/E6p and R6p/cRGD-E6p at P/N/C ratios of 1/8/1 and 1/8/2 showed the most notable and statistically significant differences in efficacy compared to the control carriers. The enhanced efficiency observed in these complexes compared to the control ones can be attributed to the overall superior performance of these peptide carriers. The level of transfection efficiency decreased as the amount of polyglutamic acid increased in the composition of ternary complexes. The decrease was apparent when comparing it to DNA/PEI and DNA/Turbofect complexes. Therefore, in a serum-free environment, the reduced transfection effectiveness of ternary polyplexes formed with a higher C/P ratio can be attributed to the depletion of positive charge from the polyplexes. Consequently, this diminishes the impact of interactions between the polyplexes and the negatively-charged proteoglycans on the cell membrane, resulting in a decrease in intracellular DNA release [60,71]. Furthermore, we have not discovered any significant statistical variations between non-modified and cRGD-modified polyplexes.

**Figure 6:**
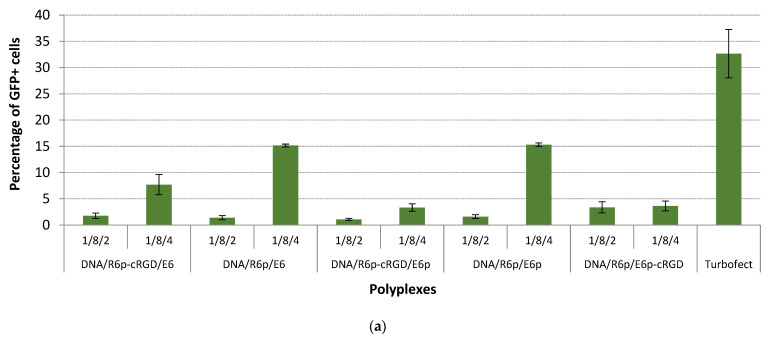

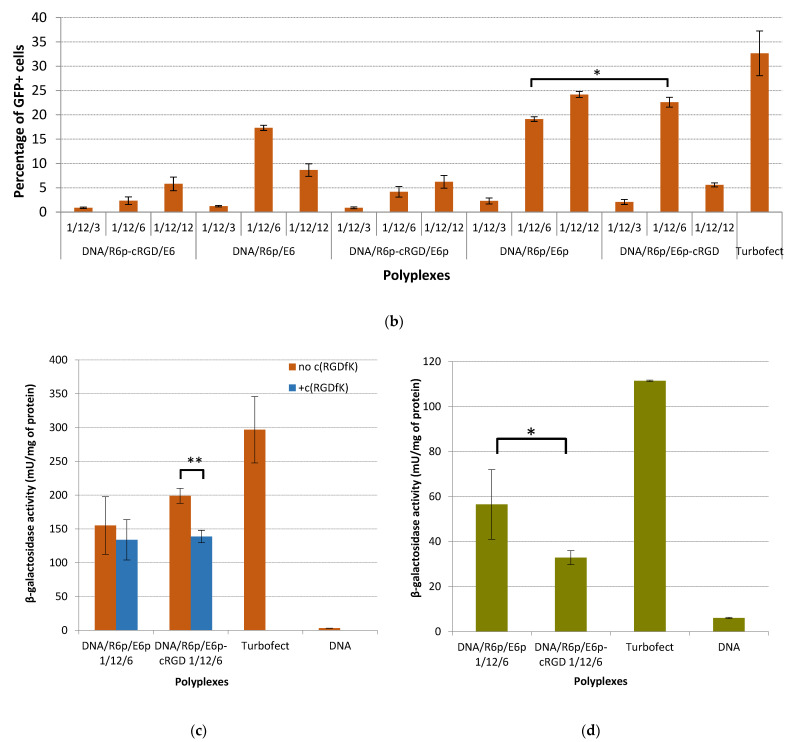
Transfection efficacy studies in the PANC-1 cells (**a**–**c**) and HEK293T cells (**d**) performed in serum-supplemented medium: (**a**,**b**) the cells were transfected with complexes of the pEXPR-IBA5-eGFP plasmid with DNA/R6p and DNA/R6p-cRGD complexes at charge ratios of 8/1 (**a**) and 12/1 (**b**), ternary polyplexes with different C/P ratios with Turbofect/DNA polyplexes as a control; (**c**) the cells were transfected in the presence of the free cyclo(RGDfK) ligand with ternary DNA/R6p/E6p and DNA/R6p/E6p-cRGD complexes at P/N/C ratio of 1/12/6, naked pCMV-lacZ plasmid and Turbofect/DNA polyplexes used as controls; (**d**) the cells were transfected with ternary DNA/R6p/E6p and DNA/R6p/E6p-cRGD complexes at P/N/C ratio of 1/12/6, naked pCMV-lacZ plasmid and Turbofect/DNA polyplexes used as controls. Values are the mean ± SD of the mean of triplicates. * *p* < 0.05, ** *p* < 0.01.

Furthermore, we conducted transfection experiments in a medium supplemented with serum, which more accurately mimics the in vivo environment, and we analyzed the activity of beta-galactosidase (Figure 5b and Figure 6c). The experiments were carried out for DNA/R6p and DNA/R6p-cRGD formed at 8 to 1 (Figure 5b) and 12 to 1 (Figure 5c) of N/P ratio, with the addition of polyglutamic acid-rich polymers at different C/P ratios. The transfection efficiency of uncoated DNA/R6p and DNA/R6p-cRGD complexes was compared to that of naked DNA in the presence of serum. Furthermore, the overall rate of transfection observed in the polyplexes studied was comparable to, and even lower than, that of Turbofect/DNA complexes. The reason for not using PEI/DNA polyplexes in the experiments was due to their low effectiveness in a medium containing serum [72]. Ternary DNA/R6p/E6p and DNA/R6p/E6p-cRGD polyplexes with a low amount of PGA (with a C/P ratio of 8/1) may potentially be bound by serum proteins as a result of their positive zeta-potential (Figure 4b). The ternary complexes with a higher concentration of PGA are most likely to exhibit increased resistance to serum inhibition due to their reduced tendency for opsonization [33]. Based on our data, we have identified that the complexes formed with P/N/C ratios of 1/8/2 and 1/8/4 exhibit the highest transfection efficiency among complexes with an N/P ratio of 8/1. The anionic coating of these complexes not only eliminates the positive charge but also enables the efficient release of DNA, as shown in Figure 2a. Polyplexes prepared with higher C/P ratios displayed a notably negative charge of approximately −30 mV, resulting in lower transfection efficiency (as shown in Figure 4b and Figure 5b). This could possibly be attributed to reduced interaction with the cell membrane and inadequate unpacking of the DNA within the cells.

The ternary polyplexes, with a N/P ratio of 12 to 1, showed high transfection efficiency in most cases. Their activity was comparable to that of Turbofect/DNA complexes. The complexes prepared with matrix-polymerized PGA at a P/N/C ratio of 1/12/24 were found to be ineffective (Figure 5c). We propose that the intracellular DNA release can be inefficient due to another type of PGA polymerization that happens at the same time as DNA complexation. In previous experiments, we have observed that DNA/R6p/E6 and DNA/R6p-cRGD/E6 polyplexes possess a significantly greater resistance to polyanion treatment (Figure 2b). In summary, the low transfection efficiency of some PGA-coated polyplexes can be attributed to both the insufficient release of DNA and the repulsion from the cellular membrane. Furthermore, when using a serum-containing medium, we did not observe any significant statistical differences in beta-galactosidase activity between the non-modified and cRGD-modified polyplexes (Figure 5b,c).

The transfection efficiency of the polyplexes in serum-supplemented medium at optimal P/N/C ratios was additionally proved by using eGFP as a reporter gene. Herein, the transfection efficacy in PANC-1 cells mediated by the ternary complexes was evaluated by flow cytometry analysis and estimated as a percentage (%) of transfected cells (Figure 6a,b). The obtained results slightly differed from the previous ones. The DNA/R6p/E6 and DNA/R6p/E6p complexes exhibited remarkable effectiveness, especially when formed at optimal P/N/C ratios. In particular, the complex formed at a ratio of 1/8/4 resulted in a substantial increase of 15% in GFP-positive PANC-1 cells percentage. Additionally, the DNA/R6p/E6 complex formed at a ratio of 1/12/6, as well as the DNA/R6p/E6p complex formed at ratios of 1/12/6 and 1/12/12, showed promising results with an increase in GFP-positive PANC-1 cells percentage ranging from17% to 24%. Remarkably, the DNA/R6p/E6p-cRGD complex, formed at a 1/12/6 ratio, demonstrated an even higher increase in the number of GFP-positive PANC-1 cells in comparison with ligand-free corresponding polyplex (*p* > 0.05). In general, the preparation of ternary polyplexes using PGA-rich polypeptides formed by oxidative polycondensation leads to more effective results, compared to those obtained with matrix-polymerized PGA. It is worth noting that the polyplexes DNA/R6p/E6p, with ratios of 1/12/6 and 1/12/12, as well as DNA/R6p/E6p-cRGD at 1/12/6, demonstrated remarkable effectiveness. These polyplexes had sizes ranging from 900 to 2300 nm and exhibited ʐ-potential values that ranged from +3 to −19 mV (Figure 4). It was widely believed that smaller DNA polyplexes should have higher transfection efficacy because they would be more efficiently taken up by cells [73]. However, several studies have shown that larger DNA complexes are more effective at transfecting cells in culture, compared to smaller ones [35,74,75,76]. It is hypothesized that the surface charge of certain ternary DNA polyplexes may not be sufficiently high to effectively stabilize the small particles, leading to the formation of larger aggregates [77]. To gain insight into this phenomenon, we conducted an analysis of four formulations using transmission electronic microscopy (TEM). Figure S2 shows that there are large aggregates ranging in size from 500 to 1000 nm in the TEM micrographs obtained. However, it is important to note that the observed aggregates are actually made up of significantly smaller nanoparticles, each measuring less than 50–100 nm in size. The discovery provides an explanation for the significant transfection effectiveness observed in the studied polyplexes. Large aggregates of polyplexes have the potential to interact with cell membranes and, as a result, some of these complexes can be taken up by the cells. Similar findings were obtained in our previous study of peptide-based polyplexes combined with anionic magnetic nanoparticles [22]. Another important discovery emerged from the transfection studies. The statistical difference observed between unmodified DNA/R6p/E6p and cRGD-modified DNA/R6p/E6p-cRGD ternary polyplexes, when using a P/N/C ratio of 1/12/6, in αvβ3 integrin-positive PANC-1 cells, indirectly validates the specificity of gene transfer with the ligand-modified complexes (Figure 6b).

To demonstrate the specificity of the ternary DNA/R6p/E6p-cRGD polyplexes for αvβ3 integrin-positive cells, the cells were first treated with the free ligand, namely the cyclo(RGDfK) peptide. In the presence of a 10-fold excess of cyclo(RGDfK), PANC-1 cells were transfected with DNA/R6p/E6p and DNA/R6p/E6p-cRGD complexes. The P/N/C ratio used was 1/12/6 (Figure 6c). The efficacy of the DNA/R6p/E6p-cRGD polyplexes was reduced by 30% as a result of the cell pre-treatment by free ligand. The presence of the cyclo(RGDfK) peptide did not cause any statistical change in the transfection efficiency of DNA/R6p/E6p polyplexes. The results obtained clearly prove the crucial role of the cRGD ligand in transfecting cells using polyplexes through targeted αvβ3 integrin binding. The transfection efficacy of DNA/R6p/E6p-cRGD complexes proved remarkably high during the competition study, primarily because of their ability to undergo non-specific uptake through absorptive endocytosis. In order to further demonstrate the targeted DNA delivery, we conducted transfection experiments on αvβ3 integrin-negative 293T cells (Figure 6d). We found that the DNA/R6p/E6p-cRGD polyplexes in these cells exhibited a 40% lower effectiveness compared to the non-specific DNA/R6p/E6p complexes. Therefore, these results demonstrate the specificity of ligand-modified complexes for delivering DNA to αvβ3-positive cells in a targeted manner.

### 2.7. Measurement of DNA Uptake

The effectiveness of DNA-polyplexes in targeting specific cells can be further demonstrated through studies of cellular uptake. The lower transfection efficiency of the complexes cannot be solely attributed to the lower cellular uptake efficiency, as it is a more complex process. Nevertheless, it can provide insights into the efficiency of cellular binding and penetration. The cell uptake is influenced by various factors, including particle size, zeta-potential, and the interaction between ligands and receptors [78]. Furthermore, the negative PGA in the polyplex composition may decrease their ability to interact with the negatively charged cell membrane and impede absorptive endocytosis. Nevertheless, the cRGD ligand possesses the capability to significantly enhance uptake in cells that express the αvβ3 integrin.

**Figure 7:**
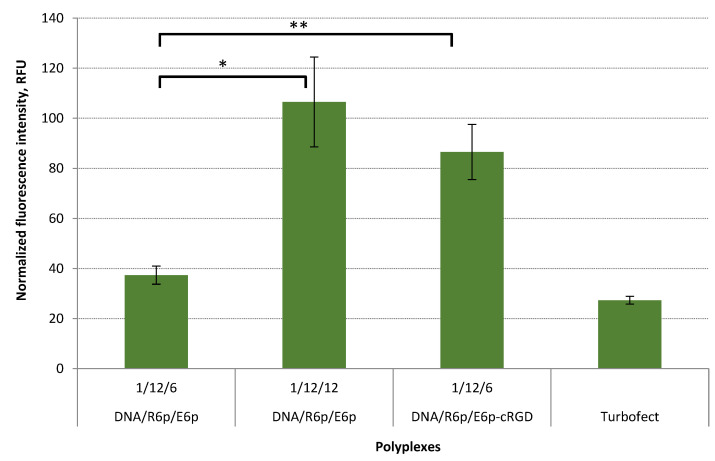
Cellular uptake in serum-supplemented medium of DNA/R6p/E6p and DNA/R6p/E6p-cRGD polyplexes in PANC-1 cells using YOYO-1-labeled pDNA. Values are the mean ± SD of the mean of triplicates. * *p* < 0.05, ** *p* < 0.01.

We utilized DNA/R6p/E6p polyplexes prepared at P/N/C ratios of 1/12/6 and 1/12/12, as well as DNA/R6p/E6p-cRGD polyplexes at a ratio of 1/12/6. These polyplexes contained fluorescently-labeled plasmid DNA, enabling us to conduct cellular uptake analysis. The labeled DNA uptake was measured by flow cytometry. A high level of normalized fluorescence intensity in the cells indicated that the studied DNA complexes were successfully taken up in serum-supplemented conditions. By increasing the quantity of PGA, there was a noticeable enhancement in the uptake of polyplexes without cRGD ligand. Furthermore, the DNA/R6p/E6p-cRGD complexes demonstrated a substantially higher intracellular uptake efficiency of 2.3-fold, compared to the DNA/R6p/E6p complexes (Figure 7). The obtained results are in line with our previous studies, confirming that the inclusion of cRGD in the polyplexes has a significant impact on the uptake level in αvβ3 integrin-positive PANC-1 cells [19,21].

### 2.8. In Vitro Suicide Gene Therapy of Uterine Leiomyoma

**Figure 8:**
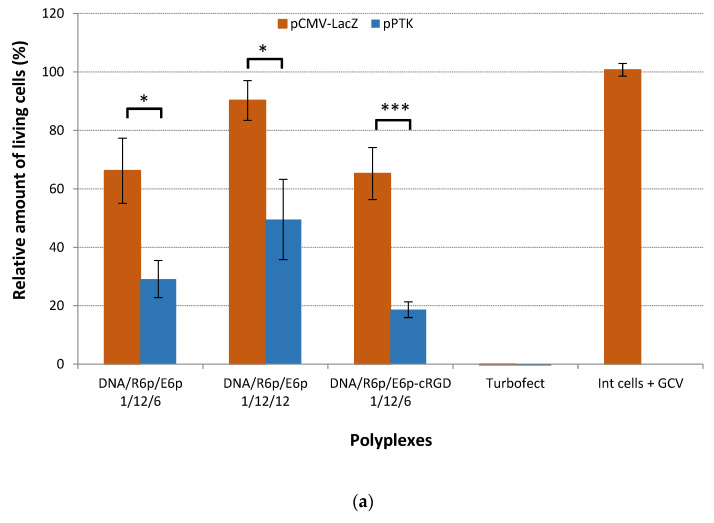

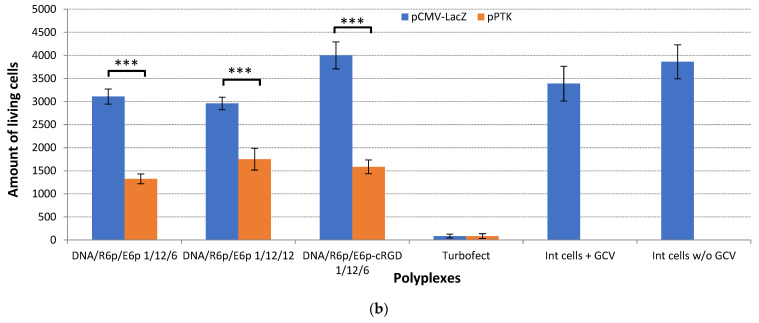
Relative UL cells viability (**a**) and amount of living UL cells (**b**) after HSV-1 thymidine kinase expression and GCV treatment. Values are the mean ± SD of the mean of triplicates. * *p* < 0.05, *** *p* < 0.001 compared to pCMV-lacZ-complexes.

In this study, we sought to utilize our advanced ternary polyplexes for the targeted treatment of UL through suicide gene therapy. In order to replicate the in vivo conditions as accurately as possible, we transfected primary leiomyoma cells with the most effective DNA/R6p/E6p polyplexes formed at ratios of 1/12/6 and 1/12/12, as well as DNA/R6p/E6p-cRGD polyplexes at a ratio of 1/12/6, in a serum-supplemented medium. The study evaluated the impact of suicide on cellular activity by utilizing Alamar blue dye (Figure 8a) to assess proliferative activity, and the Trypan blue method (Figure 8b) to measure the number of living leiomyoma cells. The effectiveness of suicide gene therapy using pPTK plasmid-carrying polyplexes was proven after four days of GCV treatment when compared to those containing pCMV-lacZ. We observed a decrease in the cells’ proliferative activity by 2.3 and 1.8 times after transfection with pPTK/R6p/E6p polyplexes, in comparison to the ones containing pCMV-lacZ, at P/N/C ratios of 1/12/6 and 1/12/12, respectively. We observed a remarkable 3.5-fold decrease in the proliferation of leiomyoma cells when using ligand-modified complexes DNA/R6p/E6p-cRGD. This result proved to be significantly more effective compared to the appropriate control polyplexes. Cell viability was significantly reduced to 29% following suicide gene therapy using cRGD-modified polyplexes, in comparison to the control pCMV-lacZ-containing complexes (Figure 8a). The reason for the higher effectiveness of pPTK/R6p/E6p-cRGD polyplexes in delivering the suicide gene to leiomyoma cells can be attributed to the elevated levels of αvβ3 integrins on these specific cells [19]. Furthermore, the obtained results provided additional evidence regarding the specificity of cRGD-modified ternary polyplexes.

The significant reduction in cell viability following suicide gene therapy, as compared to transfection efficiency illustrated in Figure 6b, can possibly be elucidated by the occurrence of the “bystander effect”. The cytotoxicity elicited by polyplexes generated using the pCMV-lacZ plasmid accounted for 70–90% of the viability of unaffected cells. Remarkably, this level of cytotoxicity greatly surpassed that of Turbofect-based complexes. The DNA/Turbofect complexes, however, proved unsuitable for research involving primary leiomyoma cells due to their excessive toxicity. Furthermore, it is worth noting that we did not observe a notable reduction in the proliferative activity of cells after treating them with GCV alone (Figure 8a). This finding strongly suggests that GCV at the experimental concentration does not produce any harmful effects on cells in the absence of the pPTK1 plasmid. Figure 8b shows that the Trypan blue assay yielded comparable data. The number of living cells was drastically reduced by up to 40% after transfection with pPTK/R6p/E6p-cRGD polyplexes, as compared to the control pCMV-lacZ ones. Transfection with pPTK/R6p/E6p polyplexes also induced a suicide effect, resulting in a remarkable decrease of viable cells by 43–59% compared to the control complexes. The pPTK1/Turbofect complexes exhibited remarkable cytotoxicity, leading to a significant decrease in the number of viable cells (Figure 8b). In conclusion, the obtained results confirm that the decrease in cell proliferation activity and amount of living UL cells is a direct result of transfection with pPTK1-polyplexes and GCV treatment, highlighting the significant impact of the suicide effects involved.

### 2.9. In Situ Gene Delivery of Reporter and HSV-TK Genes in UL Organotypc Model

**Figure 9:**
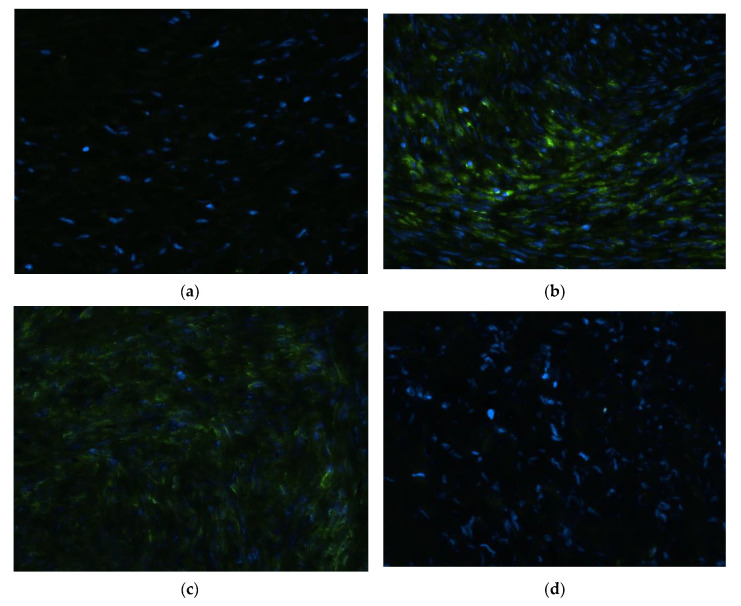
Visual appearance of myomatous nodes’ sections after the injection of DNA/R6p/E6p polyplexes formed at 1/12/6 (**a**), 1/12/12 (**b**) of P/N/C ratios and DNA/R6p/E6p-cRGD polyplexes at 1/12/6 (**c**) ratio using pEXPR-IBA5-eGFP plasmid. Intact UL tissue is used as a control (**d**). Cell nuclei stained with Hoechst 33258 show blue fluorescence; the cells with successful transfection and GFP protein production show green fluorescence. Magnification 400×.

The neutralization of cationic polyplexes’ positive charge due to extracellular matrix in uterine fibroids may hinder the successful application of gene therapy. By incorporating the negative PGA polymer, we successfully changed the charge in the ternary polyplexes. Herein, fragments of myomatous nodes were transfected with ternary DNA/R6p/E6p polyplexes formed at 1/12/6, 1/12/12 of P/N/C ratios and DNA/R6p/E6p-cRGD complexes at 1/12/6 ratio using pEXPR-IBA5-eGFP plasmid. After transfection 5–10 µm-thick sections of tissues were prepared, stained with Hoechst 33258 to mark nuclei and analyzed under a fluorescence microscope (Figure 9). The nodes injected with the DNA/R6p/E6p formed at a P/N/C ratio of 1/12/12 showed the highest level of cellular GFP protein accumulation and the largest signal area recorded especially in the injection sites (Figure 9b). GFP protein fluorescence was also observed after tissue transfection with DNA/R6p/E6p-cRGD complexes, although the signal was comparatively weaker than that observed for previous polyplexes (Figure 9c). However, transfection with the non-ligand modified DNA/R6p/E6p complexes formed at 1/12/6 ratio did not result in the appearance of green fluorescence similar to the intact tissue (Figure 9a,d).

**Figure 10:**
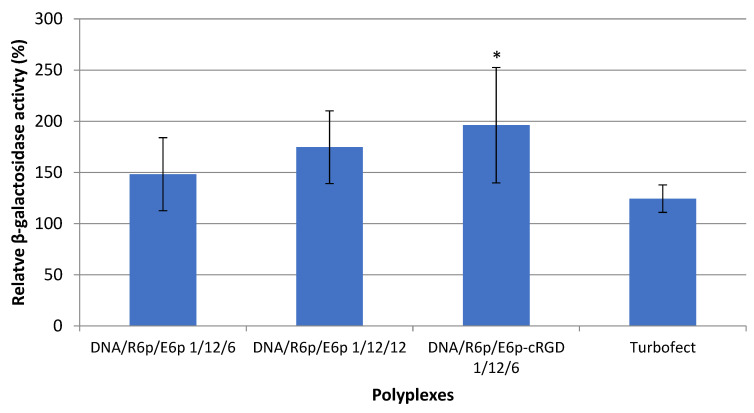
Transfection efficacy evaluation in the myomatous nodes. The tissues were transfected by DNA/R6p/E6p and DNA/R6p/E6p-cRGD polyplexes formed at P/N/C ratios of 1/12/6, 1/12/12 and 1/12/6, respectively. β-galactosidase activity is shown relatively to level in control intact UL nodes. Values are the mean ± SEM of the mean of quadruplicates.* *p* < 0.05, compared to intact tissue.

The validity of these observations was further supported by quantitatively assessing the β-galactosidase activity in the myomatous nodes following the injection of ternary complexes formed with the pCMV-lacZ plasmid (Figure 10).

Herein, the relative transfection efficiency is measured as a percentage (%) of basal level in non-transfected tissues. The results obtained showed a slight variation compared to the qualitative evaluation of GFP expression. The DNA/R6p/E6p complexes showed limited effectiveness in transfection, with no significant difference compared to the basal level. On the other hand, the transfection mediated by the DNA/R6p/E6p-cRGD complex resulted in a significant two-fold increase in β-galactosidase activity, clearly distinguishing it from the control (*p* > 0.05) (Figure 10). Based on the obtained results, it can be concluded that the incorporation of the cRGD ligand in the ternary polyplexes can provide them with a clear advantage and enhance their ability to deliver genetic material to the UL tissue.

During the last phase of the study, we assessed the therapeutic potential of the most effective formulations by using the UL organotypic model. We used DNA/R6p/E6p and DNA/R6p/E6p-cRGD polyplexes, which were formed at the optimal P/N/C ratios, to deliver the *HSV1-TK* gene to myomatous nodes. This was followed by ganciclovir treatment. The delivery of a reporter gene plasmid was employed as a means to control the experiment, in addition to utilizing intact UL nodes. The expression of pro-apoptotic genes *p53*, *Bax*, and *DAXX* was used as a reliable indicator of the effectiveness of suicide gene therapy [20,79]. Previously, gene therapeutic treatment of uterine fibroids resulted in changes of the gene expression and protein level of pro-apoptotic factors [80]. Based on this, we recently revealed increased expression of *p53*, *Bax*, and *DAXX* in UL cells after suicide gene therapy modeling, in comparison with intact cells [21]. Thus, we expected to see similar trends in the gene expression pattern in the current study.

In suicide gene therapy, the crucial aspect is the intentional disruption of DNA replication, ultimately triggering programmed cell death known as apoptosis. We conducted an assessment to determine how suicide gene therapy affects the expression levels of three pro-apoptotic transcripts. p53 plays a critical role in regulating apoptosis, participating in both the extrinsic pathway where death receptors are activated, and the intrinsic pathway involving the mitochondria [81]. It regulates the transcription activation of the Bax apoptotic factor, while the Death domain associated protein (DAXX) is specifically involved in the extrinsic pathway [82].

**Figure 11:**
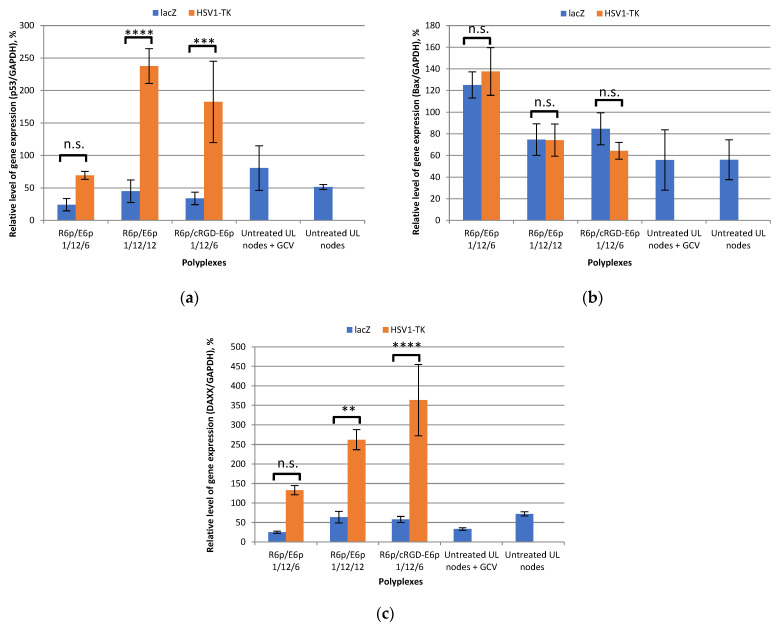
Relative expression level of pro-apoptotic genes–*p53* (**a**), *Bax* (**b**), *DAXX* (**c**) in the myomatous nodes after transfection with *HSV1*-*TK* or *lacZ* genes by DNA/R6p/E6p and DNA/R6p/E6p-cRGD polyplexes followed by ganciclovir treatment. pCMV-lacZ plasmid was used to form control polyplexes. Intact nodes and ganciclovir-treated nodes were used as negative controls. Values are the mean ± SEM (n = 5). Multiple comparisons (one-way ANOVA with Sidak correction): (**a**) n.s.—not significant (*p* = 0.3599), ****—*p* < 0.0001, ***—*p* = 0.0006; (**b**) n.s.—not significant (*p*: 0.9017; >0.9999; 0.7453); (**c**) n.s.—not significant (*p* = 0.1093), **—*p* = 0.0039, ****—*p* < 0.0001.

The efficacy of suicide gene therapy utilizing pPTK plasmid-loaded polyplexes was demonstrated following GCV treatment, in comparison to those carrying the pCMV-lacZ plasmid (Figure 11). After transfection with pPTK/R6p/E6p polyplexes at P/N/C ratios of 1/12/12, we noticed a remarkable increase in the expression levels of two pro-apoptotic genes. It was observed that the expression levels of *p53* and *DAXX* were significantly increased by 5.3 and 4.1 times, respectively, compared to the levels observed after lacZ gene delivery. We also noticed a remarkable increase of 5.4 and 6.3 times in the expression of *p53* and *DAXX*, respectively, when utilizing ligand-modified DNA/R6p/E6p-cRGD complexes. It is worth noting that the ligand-free pPTK/R6p/E6p polyplexes, formed at P/N/C ratios of 1/12/6, did not show a significant increase in the expression levels of the respective genes (Figure 11a,c). This result confirms the significant impact of cRGD ligand-modification on enhancing the transfection efficacy of the ternary polyplexes. Further understanding can be gained about the impact of an excessive anionic coating on improving transfection effectiveness, potentially in a non-specific manner.

The analysis of *Bax* gene expression after pPTK plasmid delivery reveals contrasting results. No increase in its expression level was detected, as shown in Figure 11b. Previously, the study conducted by Hassan et al. demonstrated a significant increase in the levels of *p53*, *DAXX*, and *Bax* in the UL nodes of Eker rats following Ad-DNER treatment [80]. According to the data available, DNER-gene therapy for UL nodes effectively stimulates both the internal and external pathways of apoptosis. Upon further examination of the molecular mechanisms involved in this gene therapy approach, it becomes evident that the activation of the external apoptotic pathway is a direct result of the comprehensive molecular regulation of cell death [81]. While the activation of *DAXX* results in an elevated expression level of p53-inducing genes and is in sync with p53 activity, no similar outcome was noted for *Bax* [83]. Although these genes show higher expression levels, they do not result in increased *Bax* expression. Additionally, there is no correlation in expression profile between *Bax* and *DAXX*. Our findings show a direct link between the expression data and the administration of pHSV1-TK plasmid complexes followed by ganciclovir treatment. Notably, there was a clear and consistent increase in *DAXX* and *p53* expression levels, while *Bax* expression remained largely unchanged (Figure 11).

## 3. Materials and Methods

### 3.1. Cell Lines and Organotypic Model of Uterine Leiomyoma 

Human pancreatic (PANC-1) and human kidney (293T) cell lines were obtained from the Cell Collection of the Institute of Cytology RAS (Saint-Petersburg, Russia) and cultivated under mycoplasma-free conditions at 37 °C in 5% CO_2_, as described previously [19]. Briefly, the cell lines were maintained in glutamine-supplemented DMEM medium (Biolot LLC, Saint-Petersburg, Russia; cat.no. 1.3.6.3.) with 10% St-Biol–heat-inactivated fetal calf serum (Biolot LLC, Saint-Petersburg, Russia; cat.no. 1.1.4.3.) and the addition of 0.01% of gentamicin (Biolot LLC, Saint-Petersburg, Russia; cat.no. 1.3.16.). Primary uterine leiomyoma cells and organotypic model of the disease were obtained from patients after myomectomy in the D.O. Ott Research Institute of Obstetrics, Gynecology and Reproductology (Saint-Petersburg, Russia) and maintained in completed AmnioMax medium according to recent protocol [49]. Uterine leiomyoma cells were used at passage 2, whereas organotypic model was available for experimental work up to three weeks, as recommended previously [49].

### 3.2. Peptide Carrier Preparation and Characterization

CHR_6_HC (R6), CHE_6_HC (E6), cyclic RGDyC(*Npys*) (cRGD), and cyclo(RGDfK) peptides were synthesized by NPF Verta, LLC (Saint-Petersburg, Russia) using solid-phase Boc-chemistry and stored as a dry powder at −20 °C with purity of 90–95% determined by high-performance liquid chromatography, as described earlier [21]. For R6 and E6 carriers’ preparation the peptides were dissolved in 0.1% TFA at 2 mg/mL and stored at −20 °C [47]. The R6p, E6p, R6p-cRGD, E6p-cRGD carriers were obtained by dissolving at 30 mM concentration, with 30% of DMSO, followed by oxidative polycondensation for the next 96 h, as described previously [21]. For preparation of R6p-cRGD and E6p-cRGD, the cRGD ligand was dissolved at 30 mM, with 30% of DMSO added to R6p or E6p mixture before polycondensation at the ratio of one cRGD moiety per two R6 or E6 peptides. The obtained polycondensed carriers were stored and then dissolved in distilled water at a concentration of 2 mg/mL at −70 °C. The amount of unreacted thiol groups was controlled by Ellman’s assay [84].

### 3.3. Plasmids

The pCMV-lacZ plasmid with β-galactosidase gene (under control of CMV promoter; 6821 bp size) was gifted by Prof. B. Scholte, Erasmus University Rotterdam, The Netherlands. The pEXPR-IBA5-eGFP plasmid containing green fluorescence protein (GFP) gene (under control of CMV promoter; 6176 bp size) was obtained from IBA GmbH, Göttingen, Germany. The pPTK1 plasmid with the HSV1 herpes virus thymidine kinase gene (under control of mouse rpL32 promoter; 5850 bp size) was supplied by Dr. S.V. Orlov from the Institute of Experimental Medicine, Saint-Petersburg, Russia. The plasmids were isolated according to Qiagen Plasmid Giga kit protocol (Qiagen, Hilden, Germany) and stored at −20 °C at a concentration of 0.5–1 mg/mL.

### 3.4. Preparation of Carrier/DNA Complexes

DNA/R6p and DNA/R6p-cRGD polyplexes were prepared at various N/P ratios (peptide nitrogen/DNA phosphorus ratio) in Hepes-buffered mannitol (HBM) (5% (*w*/*v*) mannitol, 5 mM Hepes, pH 7.5), and were mixed by vortexing and left at room temperature for 30 min [21,47]. Upon completion of complexes formation, negatively charged E6, E6p or E6p-cRGD carriers were added to complexes at various C/P ratios (peptide carboxyl group/DNA phosphorus ratio). E6p or E6p-cRGD- containing complexes were left for 30 min while DNA/R6p/E6 and DNA/R6p-cRGD/E6 polyplexes were incubated for the next 2 h to allow interpeptide disulphide bonds formation.

Polyethyleneimine (branched PEI 25 kDa; Sigma-Aldrich, St. Louis, MO, USA) was used at a concentration of 0.9 mg/mL (pH 7.5) as aqueous stock solution, and stored at +4 °C; the PEI/DNA charge ratio was taken as 8/1. TurboFect transfection reagent was used in accordance with the manufacturer’s recommendation—namely, 4 µL of Turbofect was taken per 1 mg of DNA (Thermo Fisher Scientific, Waltham, MA, USA).

### 3.5. DNA Binding and Relaxation of Polyplexes by Dextran-Sulfate

Carrier binding to DNA was assessed by using the ethidium bromide (EtBr) exclution assay in a Wallac 1420D scanning multilabel counter (PerkinElmer Wallac Oy, Turku, Finland) at 590 nm emission and 540 nm excitation. EtBr displacement was calculated as (F − Ff)/(Fb − Ff), where Ff and Fb are the EtBr fluorescence values in the absence and presence of DNA [85]. Dextran-sulfate (DS; Sigma, St. Louis, MO, USA) was added to the polyplexes at three-fold charge excess relative to the cationic carrier. At 0 min, 1 h, 3 h, 12 h and 24 h of incubation, EtBr fluorescence intensity was measured and the displacement was calculated.

### 3.6. Ellman’s Assay

The estimated relative amount of free thiol groups in E6 peptide bound to triple DNA-polyplexes was analyzed directly by using Ellman’s assay [84]. The aliquots of prepared complexes were mixed with solution of 5-5′-dithiobis (2-nitrobenzoic acid) (DTNB or Ellman’s reagent, Sigma, St. Louis, MO, USA) in 0.1 M phosphate buffer (pH 8.0), as previously described [47]. Absorbance was measured in Multiscan plus P reader (Labsystems, Helsinki, Finland) at 405 nm. Relative amount of free thiol groups was counted as (P − Pf)/(Pb − Pf), where Pf and Pb are the absorbance in the absence (free peptide only) and presence of DNA.

### 3.7. Size and Z-Potential Measurements, Transmission Electronic Microscopy

The size and ʐ-potential of the prepared polyplexes were measured in zetasizer NANO ZS (Malvern instruments, Malvern, UK) three times independently by using dynamic light scattering and microelectrophoresis, respectively. Electron microphotographs of the obtained ternary complexes were prepared after polyplexes negative staining with a 1% aqueous solution of uranyl acetate, and by using a transmission electron microscope Libra 120 (Carl Zeiss, Oberkochen, Germany).

### 3.8. Gene Transfer and Cytotoxicity Assays

A day before the experiment, PANC-1 or 293T cells were seeded in 48-well plates, at a density of 5.0 × 10^4^ cells per well. Complexes were prepared at the rate of 2 µg of DNA per well on an experiment day. Some initial transfections were performed under serum-free conditions; in this case before experiments medium was changed for the serum-free one and polyplexes were added for 4 h followed by 48 h incubation in serum-contained medium, as described previously [19]. Most transfections were performed in fully supplemented medium within 48h of incubation, without the medium changing. After experiments with pCMV-lacZ plasmid, cells were lysed; β-galactosidase activity (mU) was measured on the Wallac 1420D scanning multilabel counter (355 nm excitation, 460 nm emission) and then normalized by the total protein concentration in cell extracts that was determined by Bradford reagent (Helicon, Moscow, Russia) in Multiscan plus P reader with wavelength 620 nm [85]. The competition studies were performed by using free c(RGDfK) peptide with a 10-fold excess, which was added to PANC-1 cells 15 min before transfection [19]. The relative amount of GFP-positive cells was detected by flow cytometry with a BD FACS-Canto II cytofluorimeter, after the transfection of PANC-1 cells with a pEXPR-IBA5-eGFP plasmid.

The cytotoxicity of the polyplexes was evaluated on PANC-1 cells, which were seeded in a 96-well plate a day before the experiment at a density of 1.5 × 10^4^ cells per well. Complexes were prepared at the rate of 0.7 µg of DNA per well. The transfections were performed in fully supplemented medium within 24 h, followed by 3 h of incubation with Alamar Blue reagent (BioSources International, San Diego, CA, USA). The fluorescence was measured on a Wallac 1420D scanning multilabel counter (excitation 544 nm, emission 590 nm) and the relative fluorescence intensity was calculated as previously described [47].

### 3.9. Cellular Uptake of Peptide/DNA Complexes

The day before the experiment, PANC-1 cells were seeded at a density of 6 × 10^4^ cells/well in 48-well plates. Before the formation of complexes, DNA was labeled with YOYO-1 iodide (Thermo Fisher Scientific, Waltham, MA, USA) (1 molecule of the dye per 50 base pairs). Transfection was performed in a serum-supplemented medium, as described in Section 3.8. After 2 h of incubation, the cells were washed twice in 1 × PBS (pH 7.2): once with 1 M NaCl (in 1 × PBS) and again (twice in total) in 1 × PBS. Then, cells were detached, resuspended, incubated with propidium iodide solution (50 µg/mL in 1 × PBS) for 15 min to exclude dead cells, and then further analyzed by flow cytometry. The results were presented as RFU per 10,000 of living cells.

### 3.10. In Vitro Suicide Gene Therapy of Uterine Leiomyoma

Primary leiomyoma cells were seeded on 96-well plates (“Nunc”) at 1.5 × 10^4^ cells per well 24 h before the experiment. Transfections were performed in fully supplemented DMEM-F12 medium (GIBCO), with 0.7 µg of DNA (pPTK1 or pCMV-lacZ plasmid) per well. The plates were incubated for 24 h, followed by the medium changing to standard one containing 50 μg/mL of ganciclovir, before being incubated for the next 96 h. For proliferation activity measurements, the medium was replaced with the fresh one containing 10%, before the Alamar Blue reagent and the plate was incubated for 2 h. Fluorescence was then measured on a Wallac 1420D fluorimeter (wavelengths 530/590 nm) and the relative number of living cells was estimated, as described previously [20]. The cell photos were obtained by using a microscope AxioObserver Z1 (Carl Zeiss, Oberkochen, Germany) and the AxioVision program at 100× magnification. The number of living cells was counted using the Trypan blue dye exclusion method. After cell harvesting with 0.25% Trypsin-EDTA (Thermo Fisher Scientific, Carlsbad, CA, USA), cells were treated with 0.4% Trypan blue solution (Sigma-Aldrich, Munich, Germany) at a 1:1 volume ratio, and the unstained cells were counted by using a hemocytometer (MiniMedProm, Dyatkovo, Russia).

### 3.11. In Situ Gene Transfer to Myomatous Nodes

Myomatous nodes (5 cm^3^) obtained after hysteroscopic procedures were divided using scissors or a scalpel into smaller fragments (approximately 125 mm^3^) and then maintained in culture, as previously described [49]. The complexes were prepared as described in Section 3.4. The entire volume was injected into the myomatous node by using a Micro-Fine IV Insulin Syringes Needle (Becton Dickinson, Franklin Lakes, NJ, USA) to manually undertake a series of sequential injections into different sides of the node. Injected nodes were incubated in fresh fully supplemented Amniomax medium for 72 h. Transfections were performed in fresh fully supplemented Amniomax medium (GIBCO), with 5 µg of DNA (pEXPR-IBAS-eGFP or pCMV-lacZ plasmid) per node. The complexes were injected and the nodes were incubated for 72 h. Further, myomatous nodes were frozen in liquid nitrogen for an hour and stored at −70 °C. For GFP expression visualization, 5–10 µmsections were made on a cryostat Leica CM1510 (Leica Microsystems, Wetzlar, Germany). Sections were stained with Hoechst 33258 for 1 min, and then were photographed under a fluorescent microscope Leica DM 2500 (Leica Microsystems, Wetzlar, Germany) at 100× magnification. We analyzed an average of 30 sections from each of three layers (top, middle, bottom), with about 1000 µm between each UL fragment. For β-galactosidase expression studies, the tissues were triturated using homogenizer in 160 μL of the complete lysis buffer; the suspension was then transferred into 0.6 mL tubes, incubated for 40 min at +4 °C and centrifuged for 5 min at 6000 rpm, with the aim of taking away the tissue debris [85]. Detection of β-galactosidase expression was performed as described in Section 2.8.

### 3.12. RNA Extraction and RT-qPCR

Fragments of myomatous nodes (125 mm^3^) were transfected with 5 µg of pDNA (pPTK1 or pCMV-lacZ plasmid), as described in Section 3.11. After 24 h of incubation the medium was changed to a standard one containing 50 μg/mL of ganciclovir. The procedure was repeated three additional times, at a frequency of once every 24 h, beforethe incubation myomatous nodes were triturated and used for expression studies of the proapoptotic genes. Before the RNA extraction, the leiomyoma nodes were homogenized using TissueLyser LT (Qiagen, Hilden, Germany). Leiomyoma nodes were transported into 2 mL microcentrifuge tubes containing 150 µL of cold PBS and one steel bead (d = 5 mm), and the tubes were then loaded into precooled TissueLyser LT Adapter and the homogenization cycle was then run as follows: oscillation frequency of 50 Hz for 10 min; incubation at +4 °C for 10 min–repeat 2–3 times until the tissues dissociate. After homogenization, the tubes were centrifuged at 2200 rpm for 10 min, before steel beads were removed. Total RNA was then extracted with Trizol reagent (Thermo Fisher Scientific). cDNA was generated from the RNA samples by using the OT-1 Reverse Transcription kit (Syntol JSC, Moscow, Russia) in accordance with the manufacturer’s instructions. The cDNA was analysed for purity and concentration with the spectrophotometer NanoDrop 2000 (Thermo Fisher Scientific, Waltham, MA, USA), at both the 260 nm and 280 nm wavelengths. Quantitative PCR was performed with the EvaGreen PCR kit (Syntol JSC, Moscow, Russia), and the following primers were used: *Bax* forward primer 5′-TTC TGA CGG CAA CTT CAA CTG G-3′, reverse primer 5′-AGG AAG TCC AAT GTC CAG CC-3′ [86]; *p53* forward primer 5′-TAA CAG TTC CTG CAT GGG CGG C-3′, reverse primer 5′-AGG ACA GGC ACA AAC ACG CAC C-3′ [87]; *DAXX* forward primer 5′-CTG AAA TCC CCA CCA CTT CC-3′, reverse primer 5′-CTG AGCAG CTG CTT CAT CTT C-3′ [88]; and the reference gene *GAPDH* was determined using forward 5′-CGC CAG CCG AGC CAC ATC-3′, reverse 5′-CGC CCA ATA CGA CCA AAT CCG-3′ [89] primers. The values are presented as mean ± SEM of the means obtained from five independent experiments.

### 3.13. Statistical Analysis

Statistically significant differences were analyzed by applying the Mann–Whitney U-test, the Student’s *t*-test and ANOVA (using the GraphPad Prism 8 v. 8.02; GraphPad Software Inc., San Diego, CA, USA). *p* < 0.05 was considered statistically significant.

## 4. Conclusions

This study introduces an innovative approach to transfect DNA to uterine leiomyoma cells by using cRGD-ligand-decorated-polyanion-coated ternary polyplexes. The role of polyanion coating in achieving serum resistance by the ternary polyplexes is confirmed by both physicochemical and cell transfection experiments. The cells clearly demonstrated αvβ3 integrin-specific uptake of the DNA/R6p/E6p-cRGD complexes, as evidenced by successful competitive transfections with the free cyclic RGD ligand. The application of suicide gene therapy, particularly the delivery of the plasmid encoding the thymidine kinase to uterine leiomyoma cells, followed by treatment with ganciclovir, resulted in significant therapeutic outcomes. The expression of the HSV-1 thymidine kinase gene in uterine leiomyoma tissues led to a remarkable increase in the expression of the pro-apoptotic *p53* and *DAXX* genes. Our findings strongly indicate that the ternary polyplexes we have developed have immense potential as a valuable tool for implementing suicide gene therapy of uterine leiomyoma and are ready for the next stage of pre-clinical studies of the UL animal model.

## Figures and Tables

**Table 1:** Composition of monomers and carriers.

	Name	Composition
Monomers	R6	CHRRRRRRHC
	E6	CHEEEEEEHC
Ligand monomer	cRGD	C(*Npys*)RGDy |__________|
Polycondensed carriers	R6p	(CHRRRRRRHC)n
	R6p-cRGD	cRGD-(CHRRRRRRHC)n-cRGD
	E6p	(CHEEEEEEHC)n
	E6p-cRGD	cRGD-(CHEEEEEEHC)n-cRGD

## Data Availability

The data are not publicly available due to restrictions imposed by the subjects’ agreement.

## Supplementary Materials

The following supporting information can be downloaded at: https://www.mdpi.com/article/10.3390/ijms25010034/s1.
