# Supplementary material for: Serum-Resistant Ternary DNA Polyplexes for Suicide Gene Therapy of Uterine Leiomyoma
Source: Int J Mol Sci. 2023 Dec 19;25(1):34. doi: 10.3390/ijms25010034 (PMC10778803; doi:10.3390/ijms25010034)
Supplement: Supplementary file 1 [file ijms-25-00034-s001.zip › ijms-2763582-supplementary.pdf]

Supporting Information for the Article

Serum-resistant ternary DNA-polyplexes for suicide gene therapy of uterine leiomyoma

Anna Egorova [1], Sofia Shtykalova [1], Marianna Maretina [1], Svetlana Freund [1], Alexander Selutin [2], Natalia Shved [1], Sergei Selkov [2], and Anton Kiselev [1,*]

[1] Department of Genomic Medicine named after V.S. Baranov, D.O. Ott Research Institute of Obstetrics, Gynecology and Reproductology, Mendeleevskaya line 3, 199034 Saint-Petersburg, Russia; egorova_anna@yahoo.com (A.E.); sofia.shtykalova@gmail.com (S.S.); marianna0204@gmail.com (M.M.); svetafreundmax@gmail.com (S.F.); natashved@mail.ru (N.S.);

[2] Department of Immunology and Intercellular Interactions, D.O. Ott Research Institute of Obstetrics, Gynecology and Reproductology, Mendeleevskaya line 3, 199034 Saint-Petersburg, Russia; a_selutin@yahoo.com (A.S.); selkovsa@mail.ru (S.S.)

***** Correspondence: ankiselev@yahoo.co.uk; Tel.: +7-812-328-9809

Contents

Figure S1. Evaluation of the cytotoxicity after transfection of PANC-1 cells with DNA/R6p/E6p and DNA/R6p/E6p-cRGD polyplexes……….....3

Figure S2. Typical micrographs of the ternary polyplexes obtained by transmission electronic microscopy...5

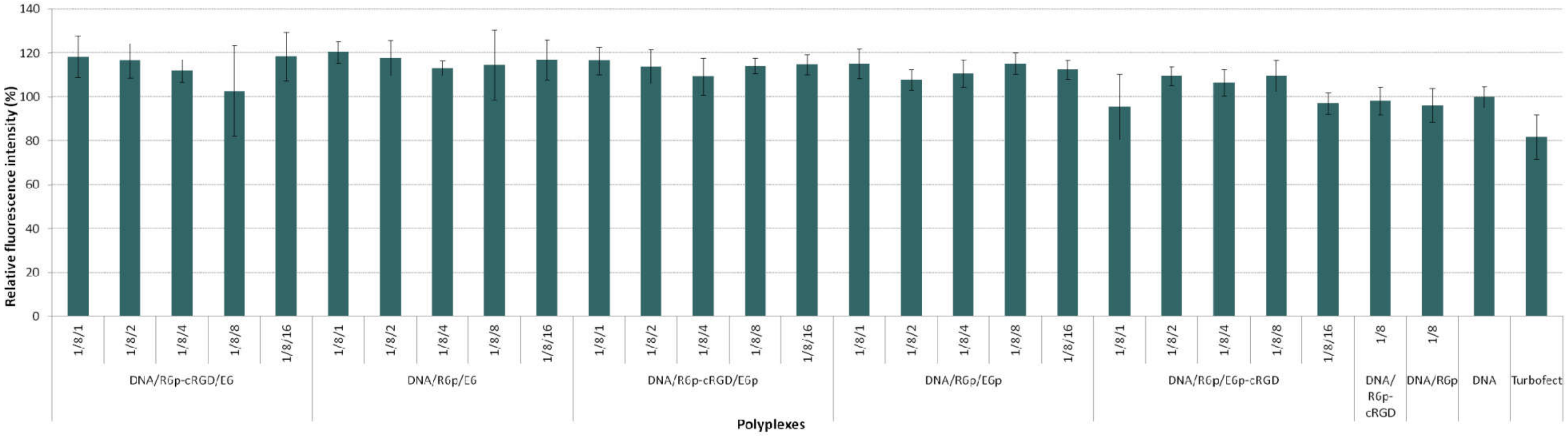
Relative fluorescence intensity (%)
Polyplexes
DNA/R6p-cRGD/E6
DNA/R6p/E6
DNA/R6p-cRGD/E6p
DNA/R6p/E6p
DNA/R6p/E6p-cRGD
DNA/R6p-cRGD
DNA/R6p
DNA
Turbofect

(a)

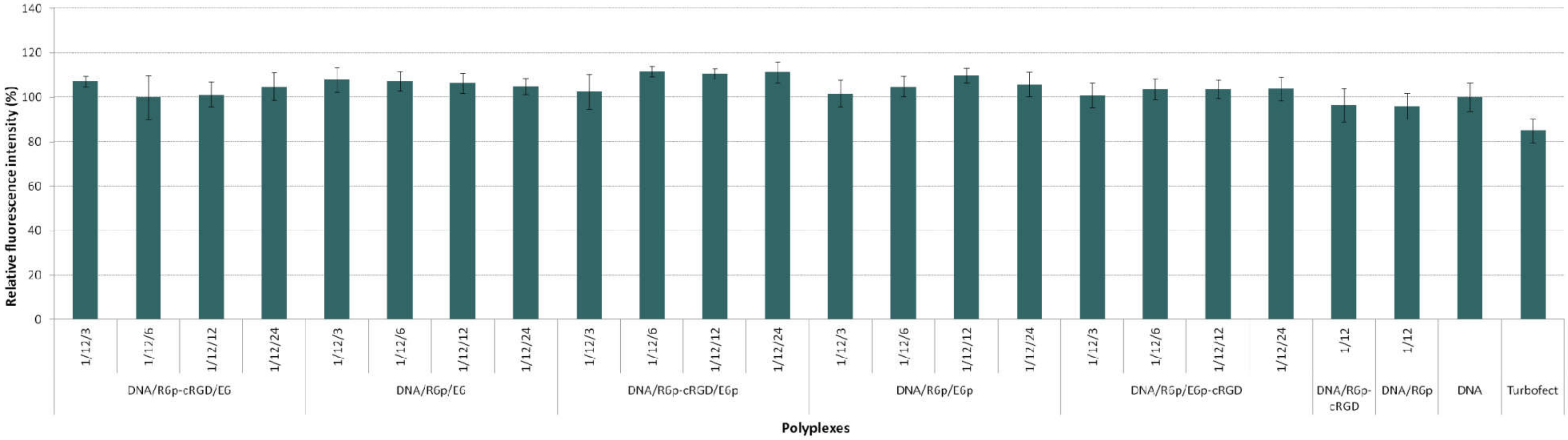
Relative fluorescence intensity (%)
Polyplexes
DNA/R6p-cRGD/E6
DNA/R6p/E6
DNA/R6p-cRGD/E6p
DNA/R6p/E6p
DNA/R6p/E6p-cRGD
DNA/R6p-cRGD
DNA/R6p
DNA
Turbofect

(b)

Figure S1. Evaluation of the cytotoxicity after transfection of PANC-1 cells with DNA/R6p/E6p and DNA/R6p/E6p-cRGD polyplexes formed at different P/N/C ratios: **(a)** N/P ratio of 8/1; **(b)** N/P ratio of 12/1. Values are the mean ± SD of the mean of triplicates.

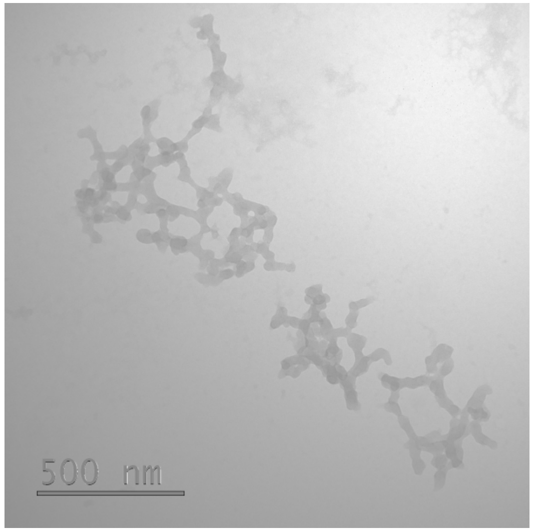

(**a**)

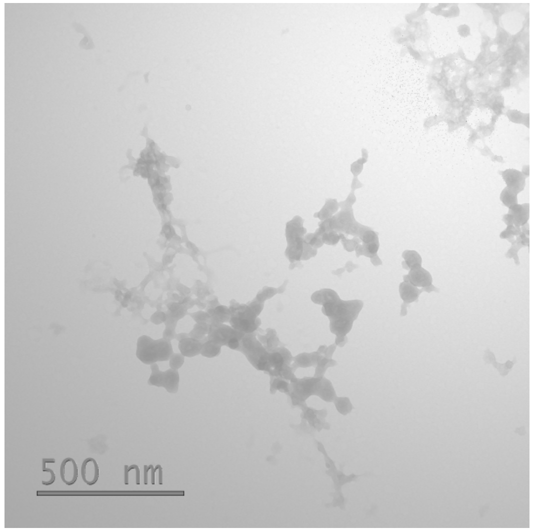

(**b**)

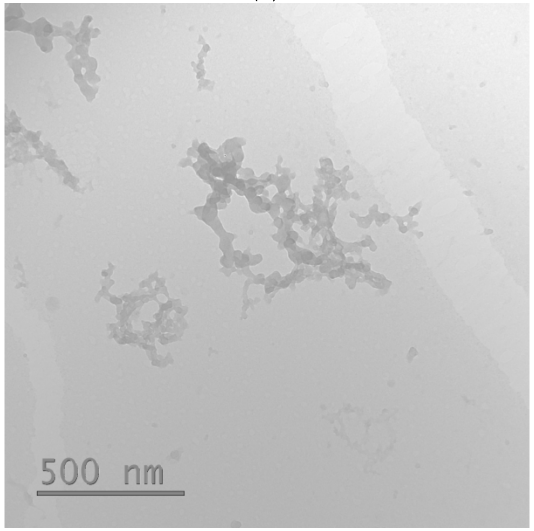

(**c**)

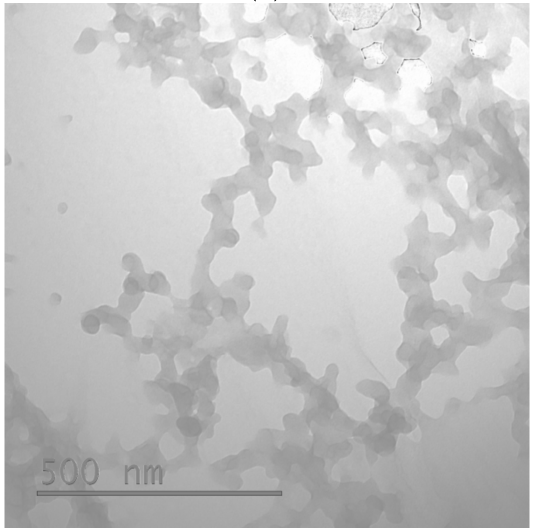

(**d**)

Figure S2. Typical micrographs of the ternary polyplexes obtained by transmission electronic microscopy: (**a**) DNA/R6p/E6p at P/N/C ratio 1/8/4; (**b**) DNA/R6p/E6p at P/N/C ratio 1/12/6; (**c**) DNA/R6p/E6p at P/N/C ratio 1/12/12; (**d**) DNA/R6p/E6p-cRGD at P/N/C ratio 1/12/6.
